# Different meanings of a three-point decline in MMSE score in Alzheimer's disease and depressive disorder

**DOI:** 10.1192/bjo.2024.732

**Published:** 2024-08-08

**Authors:** Karolina Sejunaite, Yosra Belal, Claudia Lanza, Matthias W. Riepe

**Affiliations:** Division of Geriatrics and Geriatric Psychiatry, Department of Psychiatry and Psychotherapy II, Ulm University, Ulm, Germany

**Keywords:** Alzheimer's disease, depression, cognition, biomarkers, neuropsychology

## Abstract

**Background:**

The Mini-Mental State Examination (MMSE) is a composite scale that is included in diagnostic algorithms and in procedures to assess severity of cognitive impairment and efficacy of therapeutic interventions. It is unclear, however, whether the MMSE provides information about the same deficits in different diseases.

**Aims:**

To assess patterns of MMSE scores in patients with confirmed diagnosis of Alzheimer's disease or depressive disorder.

**Method:**

We used data from a previously published cross-sectional retrospective observational clinical cohort study. The final analysis included only patients in whom biomarker analysis showed results characteristic of Alzheimer's disease (*n* = 167) and patients with depressive disorder in whom Alzheimer's disease had been ruled out by analysis of biomarkers (*n* = 69).

**Results:**

A three-point decline in MMSE score from 30 to 27 reflected impairment of memory recall in patients with Alzheimer's disease, whereas it reflected impairments in calculation and memory recall in patients with depressive disorder. A further three-point decline in MMSE score from 27 to 24 predominantly reflected additional calculation impairment in patients with Alzheimer's disease.

**Conclusions:**

Our results indicate that memory performance is the most important measure of disease severity and the main contributor to the decline in MMSE score at onset of clinical manifestation of Alzheimer's disease. In general, this suggests that memory should be the primary measure used in routine clinical care and the primary endpoint in clinical trials involving patients with Alzheimer's disease at onset of clinical manifestation. Changes in other measures of cognition should prompt consideration of possible comorbidities as a cause, rather than the impact of Alzheimer's disease itself.

Deteriorating cognition and depressive symptoms are frequent in old age and often coexist. Alzheimer's disease is the most common cause of cognitive decline in old age, and cognitive symptoms in patients with Alzheimer's disease are frequently accompanied by depressive symptoms. Likewise, depressive disorder is frequent in old age, and its symptoms are often accompanied by cognitive deterioration. Incipient dementia is the most common misdiagnosis in individuals with depressive disorder and *vice versa*. Clinical assessment and use of standardised scales do not sufficiently distinguish between Alzheimer's disease and depressive disorder. Thus, confirmation of the diagnosis of Alzheimer's disease or depressive disorder requires analysis of biomarkers.^1^

The high prevalence of cognitive impairment in old age necessitates the administration of short screening tests to capture cognitive deficits. One of the most widely used tests is the Mini-Mental State Examination (MMSE).^2^ The 1984 guidelines on diagnosis and treatment of dementia state that standardised cognitive scales, such as the MMSE, are useful for confirming a diagnosis of dementia and characterising the progression of dementia.^3^ Likewise, the MMSE may be used to appraise a patient's response to therapy.^3^ The MMSE has also been used to characterise cognitive decline in depressive disorder.^4^

Despite frequent criticism, the MMSE remains in widespread use in both routine clinical care and screening procedures for clinical trials. The MMSE is a composite scale; a total score is generated based on tasks in different cognitive domains, e.g. orientation, memory, calculation and other cognitive aspects. The same approach is used in other composite instruments to assess cognitive performance in Alzheimer's disease, e.g. the cognitive scale of the Alzheimer's Disease Assessment Scale,^5^ the Clinical Dementia Rating Scale^6^ and the Repeatable Battery for the Assessment of Neuropsychological Status.^7^

Decline in cognitive function in patients with Alzheimer's disease follows a characteristic and sequential pattern.^8^ At onset, deficits of episodic memory and spatial orientation are predominant. With spread of the disease, the severity of these symptoms increases, and further impairments accrue. This deterioration comprises executive functions, attention, working memory, visuospatial functions and further domains.^8^

Depressive disorder is characterised by depressed mood, diminished drive and anhedonia. It is well established that patients with depressive disorder are also affected by cognitive symptoms. A multitude of studies have shown that depressive disorder is associated with impairment in short-term memory, sustained and selective attention, alertness, cognitive flexibility and executive functions.^9,13^ However, the associations of the severity and pattern of cognitive deficits with the severity of the affective symptoms of depressive disorder have not been established.

Every cognitive test has a ceiling effect and a floor effect. Both occur when an independent variable (e.g. cognitive capability) no longer has an effect on a dependent variable (e.g. test performance). A test may be too easy in the early stages of a disease, resulting in a ceiling effect; conversely, it may be too difficult in the late stages, resulting in a floor effect. For instance, there is no use assessing episodic memory in patients with late Alzheimer's disease, because a floor effect is observed even with simple episodic memory tasks.

Composite scales comprise multiple tasks, and a decline in the total score reflects an overall decline across several tasks. However, some tasks may not contribute to the decline because of floor or ceiling effects. This raises the question of whether an itemised analysis of decline needs to be considered when using composite tests for the assessment of cognitive decline in different diseases.

A recent consensus paper came to the conclusion that Alzheimer's disease can be ruled out if cerebrospinal fluid biomarkers of Alzheimer's disease are negative.^14^ This implies that Alzheimer's disease diagnosis can be confirmed by biomarker analysis in patients with cognitive impairment, regardless of the presence of additional depressive symptoms. If biomarkers are positive, Alzheimer's disease needs to be diagnosed. By contrast, depressive disorder needs to be diagnosed in patients with depressive symptoms and cognitive impairment only if biomarkers are not suggestive of Alzheimer's disease. Thus, the diagnoses of Alzheimer's disease and depressive disorder can be verified in patients with cognitive deficits and depressive symptoms using biomarker analysis.

Here, we present an itemised analysis of the MMSE in patients with verified Alzheimer's disease and verified depressive disorder. This may help to improve understanding and use of the MMSE and other composite scales in complex disorders progressing over time. Specifically, we investigate whether an early decline from the maximum total score on the MMSE provides information about the same deficits in Alzheimer's disease and depressive disorder.

## Method

We performed an observational clinical cohort study using patient records from the geriatric psychiatry services of Ulm University at Bezirkskrankenhaus Günzburg. The study was a retrospective analysis of routine clinical charts. As such, no informed consent was recorded at the time of assessment. The authors assert that all procedures contributing to this work comply with the ethical standards of the relevant national and institutional committees on human experimentation and with the Helsinki Declaration of 1975, as revised in 2013**.** All procedures involving patients were approved by the Ethics Committee of Ulm University (approval 289/18).

### Study sample

The study sample and diagnostic procedures were as described previously.^1,13^ In brief, the geriatric psychiatry services of Ulm University at Bezirkskrankenhaus Günzburg serve both as a primary geriatric psychiatry service for a rural catchment area of about 650 000 people and as a university-affiliated tertiary referral centre for geriatric psychiatry.^1,13^ Demographic variables for all patients with verified diagnoses of Alzheimer's disease and depressive disorder are shown in Table 1.

**Table 1:** Demographics and neuropsychological data for patients with Alzheimer's disease and depressive disorder

	Alzheimer's disease			Depressive disorder		
MMSE score	Male/female	Age, years	GDS score	Male/female	Age, years	GDS score
		mean ± s.d.	mean ± s.d.		mean ± s.d.	mean ± s.d.
24	9/19	76.1 ± 6.6	4.7 ± 4.1	2/6	77.5 ± 9.2	9.4 ± 3.8
25	11/15	78.3 ± 5.4	3.9 ± 3.4	4/3	74.3 ± 7.3	6.7 ± 3.1
26	9/20	78.0 ± 7.1	5.9 ± 3.9	5/6	74.7 ± 6.2	6.0 ± 2.9
27	12/17	76.9 ± 6.4	5.1 ± 3.5	3/5	74.3 ± 5.8	6.6 ± 4.3
28	15/10	77.3 ± 8.0	4.4 ± 3.7	7/5	71.9 ± 9.1	6.2 ± 4.3
29	8/10	74.9 ± 7.3	5.5 ± 3.8	7/6	69.8 ± 6.9	7.2 ± 2.7
30	5/6	77.0 ± 8.5	4.9 ± 4.4	5/5	72.0 ± 8.9	7.7 ± 5.0

MMSE, Mini Mental State Examination; GDS, Geriatric Depression Scale.

### Clinical scales

The MMSE^2^ is widely used to obtain an overview of global cognitive functioning. It comprises questions on orientation, registration, short-term memory, language use, comprehension and basic motor skills. The score ranges from 0–30, where lower scores indicate more severe cognitive deficits. The short version of the Geriatric Depression Scale^15,16^ is a 15-item questionnaire to assess symptoms of depression. Participants are asked to answer each item with ‘yes’ or ‘no’. A score above 5 indicates depression.^17^

### Statistical analyses

All statistical data analyses were carried out using SPSS 25.0 for Windows (Armonk, NY, 2017). Group comparisons for dichotomous variables were performed using χ^2^-tests. The normality of distribution for all other variables was determined with the Kolmogorov–Smirnov test. As not all parameters were normally distributed, differences between groups were analysed using the non-parametric Mann–Whitney U-test.

Alzheimer's disease begins with impairment of memory, followed by impairment of executive and visuospatial functions; praxia and other cognitive functions are intact in the early stages of the disease. Given this background, and to avoid the probability of increasing type II errors, we did not correct for multiple comparisons.

## Results

**Fig. 1:**
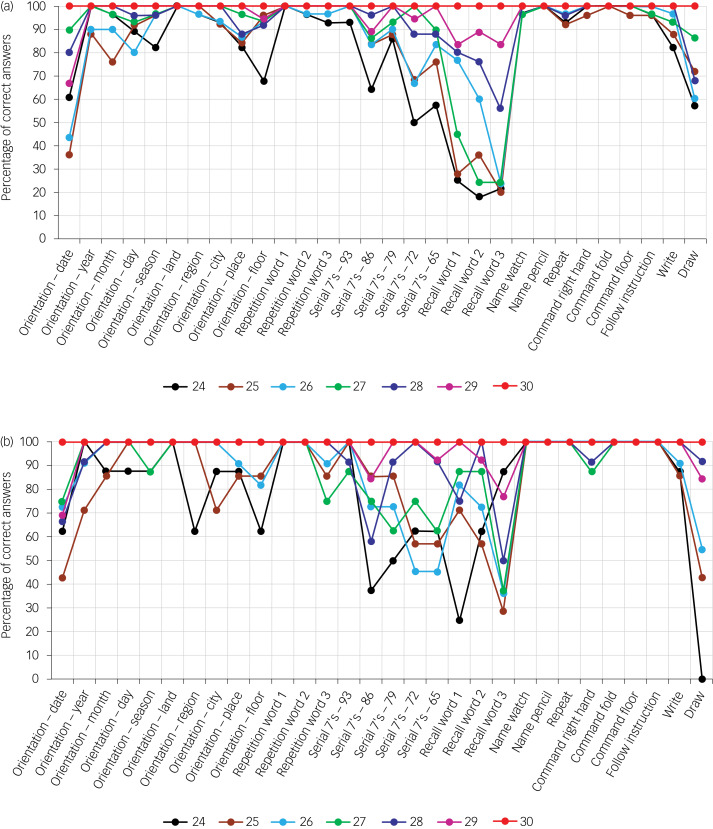
Probabilities of answering each item of the Mini-Mental State Examination (MMSE) correctly for patients with different total scores (MMSE total score is indicated in different colors below the figure), among patients with (a) verified Alzheimer's disease and (b) verified depressive disorder.

Fig. 1 shows the cognitive performance on each item of the MMSE of patients with verified Alzheimer's disease or depressive disorder and total MMSE scores of 24 to 30. The distribution of MMSE scores was similar in patients with Alzheimer's disease and those with depressive disorder (d.f. = 6; χ^2^ = 10.576; *P* = 0.102).

The predominant impairment in patients with Alzheimer's disease was recall of memory items. An itemised analysis showed that impairment of recall was greater in patients with Alzheimer's disease for all items (Table 2). Likewise, orientation with respect to day was more impaired in patients with Alzheimer's disease (Table 2). Impairment was more disseminated in patients with depressive disorder than in those with Alzheimer's disease and comprised orientation, calculation and recall. The relative difficulty of recalling words was more pronounced in patients with Alzheimer's disease than in patients with depressive disorder. Other tasks, e.g. naming, had a ceiling effect in patients with Alzheimer's disease and depressive disorder over the range of MMSE scores from 24 to 30.

**Table 2:** Percentages of whole groups of patients with Alzheimer's disease and depressive disorder who scored positive on each of the items of the Mini-Mental State Examination (group differences are set in bold; group differences that cannot be determined are denoted by n.d.)

	Alzheimer's disease (*n* = 166)	Depressive disorder (*n* = 69)		
	*n* (%)	*n* (%)	Chi-squared	*P* (exact)
Orientation – date	108 (65.1)	49 (71.0)	0.779	0.234
Orientation – year	160 (96.4)	65 (94.2)	0.570	0.331
Orientation – month	155 (93.4)	67 (97.1)	1.296	0.209
**Orientation – day**	**152 (91.6)**	**68 (98.6)**	**3.979**	**0.035**
Orientation – season	157 (94.6)	67 (97.1)	0.695	0.324
Orientation – land	166 (100)	69 (100)	n.d.	n.d.
Orientation – region	164 (98.8)	66 (95.7)	2.312	0.152
Orientation – city	160 (96.4)	66 (95.7)	0.071	0.522
Orientation – place	149 (89.8)	66 (95.7)	2.174	0.108
Orientation – floor	149 (89.8)	63 (91.3)	0.132	0.462
Repetition word 1	166 (100)	69 (100)	n.d.	n.d.
Repetition word 2	164 (98.8)	69 (100)	0.838	0.498
Repetition word 3	163 (98.2)	65 (94.2)	2.685	0.115
Serial 7's – 93	164 (98.8)	67 (97.1)	0.836	0.337
**Serial 7's – 86**	**140 (84.3)**	**51 (73.9)**	**3.480**	**0.049**
**Serial 7's – 79**	**154 (92.8)**	**57 (82.6)**	**5.489**	**0.020**
Serial 7's – 72	130 (78.3)	55 (79.7)	0.057	0.480
Serial 7's – 65	137 (82.5)	52 (75.4)	1.591	0.140
**Recall word 1**	**96 (57.8)**	**55 (79.7)**	**10.158**	**<0.001**
**Recall word 2**	**85 (51.2)**	**58 (84.1)**	**22.083**	**<0.001**
**Recall word 3**	**64 (38.6)**	**42 (60.9)**	**9.802**	**0.001**
Name watch	165 (99.4)	69 (100)	0.417	0.706
Name pencil	166 (100)	69 (100)	n.d.	n.d.
Repeat	160 (96.4)	69 (100)	2.559	0.121
Command right hand	165 (99.4)	67 (97.1)	2.039	0.207
Command fold	166 (100)	69 (100)	n.d.	n.d.
Command floor	165 (99.4)	69 (100)	0.417	0.706
Follow instruction	163 (98.2)	69 (100)	1.263	0.351
Write	155 (93.4)	66 (95.7)	0.452	0.369
Draw	123 (74.1)	49 (71.0)	0.236	0.370

**Fig. 2:**
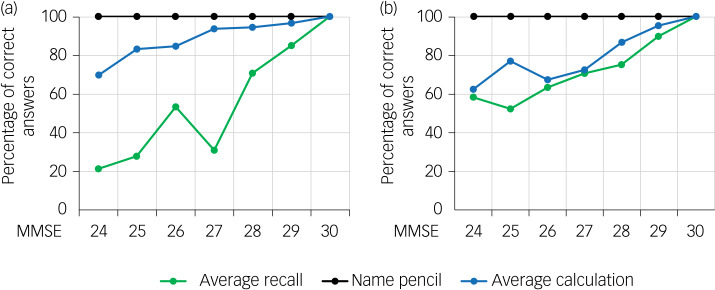
Probability of correct answers for recall, calculation and naming a pencil in patients with (a) verified Alzheimer's disease and (b) verified depressive disorder, for different total scores on the Mini-Mental State Examination (MMSE).

The relative difficulty of the calculation tasks varied with the MMSE score of the participants in a disease-specific manner. In patients with Alzheimer's disease with MMSE scores of 27 and below, memory was severely impaired, whereas calculation was much less impaired. By contrast, both calculation and recall of memory items were moderately impaired in patients with depressive disorder with MMSE scores of 27 and above (Fig. 2). Although the overall trend for all cognitive scores was clear for both Alzheimer's disease and depressive disorder patients, there were numerical deviations in the results for average recall in patients with Alzheimer's disease and MMSE total scores of 26 and 27 and those for average recall and average calculation in patients with depressive disorder and MMSE total score of 25. This may have resulted from the small group size or comorbidities in these patients (see ‘Limitations’).

Depending on whether the diagnosis was Alzheimer's disease or depressive disorder, the loss of three points on the MMSE had different implications whether the decline was from 30 to 27 or from 27 to 24. In patients with Alzheimer's disease, the three-word recall score declined by 69.0 ± 11.9% (*P* = 0.011) with a decline in MMSE score from 30 to 27, whereas it declined by 9.6 ± 9.0% (*P* = 0.002) with a decline in MMSE score from 27 to 24. The decline in calculation score (6.2 ± 6.2%) was smaller than that of the memory recall score in patients with Alzheimer's disease showing a decrease in MMSE from 30 to 27 (*P* < 0.001), whereas the declines in these two scores (23.8 ± 18.1%) were similar in patients with an MMSE score between 24 and 27.

**Fig. 3:**
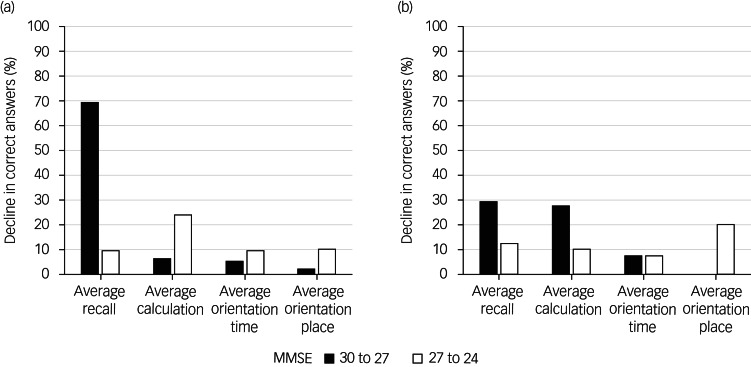
Percentage change in recall, calculation, and spatial and temporal orientation in patients with (a) verified Alzheimer's disease and (b) verified depressive disorder, for different total scores on the Mini-Mental State Examination (MMSE).

By contrast, in patients with depressive disorder, the impairments in calculation (27.5 ± 10.5%) and memory recall (29.2 ± 28.9%) were similar (*P* = 0.91) in patients with MMSE scores of 30 to 27 and those with MMSE scores between 27 and 24 (calculation: 10 ± 18.5%; memory recall: 12.5 ± 57.3%; *P* = 0.93) (Fig. 3).

## Discussion

Both cognitive and affective symptoms are present in patients with Alzheimer's disease and in those with depressive disorder. Even today, differential diagnosis of Alzheimer's disease and depressive disorder is often based on clinical symptoms and assessment with composite scales. However, reliable differential diagnosis of Alzheimer's disease and depressive disorder requires the use of biomarkers.^1^

In the present study, we analysed results only from patients with verified diagnoses of Alzheimer's disease or depressive disorder; this was an important strength of the study. However, there were also some limitations. The overall group size was fairly large, but less so after it was split up into groups corresponding to each total score from 24 to 30 on the MMSE. Moreover, comorbidities and drug use were not analysed in the present study. Alzheimer's disease is known to begin with impairment of memory, followed by impairment of executive and visuospatial functions; praxia and other cognitive functions are intact in the early stages of the disease. Given this established background, we did not correct for multiple comparisons, in order to avoid the probability of increasing type II errors.

Our results suggest that some tasks of the MMSE are predominantly impaired in patients with mild cognitive deterioration due to verified Alzheimer's disease and in those with verified depressive disorder over a range of total MMSE scores from 30 down to 24. It is well established that episodic memory performance is decreased in patients with Alzheimer's disease at onset of clinical disease.^8^ The present results show that this decline can be observed even using the task of recalling three words in the MMSE. Decline in this task is much more pronounced in patients with Alzheimer's disease than in patients with depressive disorder over the whole range of severity from MMSE score of 30 down to a MMSE score of 24. This observation is in good harmony with a past functional imaging study of our group showing that mediotemporal brain structures crucial for memory storage are more impaired in Alzheimer's disease than in depressive disorder.^18^ The observations in the present study are also in good harmony with general clinical knowledge and a previous report in the literature in patients with unverified diagnosis of Alzheimer's disease in more advanced stages of disease.^19^ Not until memory is impaired, other functions such as calculation and temporal and spatial orientation are also decreased.

Many studies have demonstrated that cognitive testing does not allow reliable differential diagnosis of Alzheimer's disease and depressive disorder or the distinction thereof in an individual patient.^1,13,20,21^ Nevertheless, there are group differences between the cognitive profiles of patients with Alzheimer's disease and those with depressive disorder. The present study demonstrates that these group differences can be observed even using the MMSE. Both calculation and memory recall are impaired in patients with depressive disorder, consistent with reports that brain structures crucial for executive functions are impaired in depressive disorder.^22,23^

The MMSE and other composite scales have also been used to monitor longitudinal changes in cognition in patients with Alzheimer's disease. Previous studies in untreated patients have shown an average decline in MMSE score of one to two points per year in patients with an initial MMSE score of 20 and above.^24^ Less is known about the pattern of cognitive decline as assessed by MMSE declines in patients with Alzheimer's disease and those with depressive disorder (MMSE ≥24). The present study demonstrates that at onset of clinical disease, the deficits in patients with Alzheimer's disease result predominantly from deficits in word recall, whereas both word recall and calculation are affected in patients with depressive disorder with mild cognitive deficits.

These results question the usefulness of composite scales for monitoring patients over longer periods of time. With long observation times, patients may start in the ceiling phase for some tasks, leading to underestimation of the change in disease severity. Likewise, patients may enter in the floor phase for some tasks, also causing underestimation of any change. Moreover, different tasks are represented by different scores in composite scales. Thus, the effect sizes of changes in total score on a composite scale or treatment effects measured with a composite scale are subject to an interaction of time and severity of disease.^25^

Appraising the efficacy of drugs is important owing to limited funds of public health systems. A clear understanding of the above results may help to prevent inadequate selection of endpoints of clinical trials. The results of the present study suggest that at clinical onset of Alzheimer's disease, progression of the disease should be assessed with measures of memory. The dynamic variability of the MMSE for patients with Alzheimer's disease at clinical onset of disease is about three, compared with a total score of 30. Changes on items other than word recall in the MMSE or other composite scales should probably not be considered to be due to Alzheimer's disease; rather, they should trigger consideration of comorbidities such as depressive disorder or vascular disease.

The brevity of the word list in the MMSE impedes its use in assessing treatment response. A more promising approach seems to be to assess word recall using long word lists, as in the California Verbal Learning Test or the Rey Auditory Verbal Learning Test. These tests also show more potential to be used as the primary endpoints of clinical trials in patients at onset of clinical Alzheimer's disease, rather than composite scales.

We conclude that memory performance is the most important measure of disease severity and decline in MMSE score at onset of the clinical manifestation of Alzheimer's disease. In general, this suggests that memory should be the primary measure in routine clinical care and the primary endpoint in clinical trials in patients with Alzheimer's disease at the onset of clinical manifestation. Changes in other measures of cognition should prompt consideration of possible comorbidities as a cause, rather than the impact of Alzheimer's disease itself.

## Data Availability

The data supporting the findings of this study are available within the article.
